# Who Did It? Sleep, Source Memory, and Action Memory in Human 4-Year-Olds

**DOI:** 10.3390/bs16091578

**Published:** 2026-09-04

**Authors:** Carolin Konrad, Rebekka Heinen, Neele Hermesch, Sarah Gerson, Jessica Sommerville, Annika Fricke, Jonas Moß, Marie-Sophie Macioszek, Alica Tabea Weinert, Sabine Seehagen

**Affiliations:** 1 Developmental Psychology, Faculty of Psychology, Ruhr University Bochum, 44801 Bochum, Germany; carolin.konrad@rub.de (C.K.); rebekka.heinen@rub.de (R.H.); neele.hermesch@rub.de (N.H.); annika.fricke@rub.de (A.F.); jonas.moss@rub.de (J.M.); marie-sophie.macioszek@rub.de (M.-S.M.); alica.weinert@rub.de (A.T.W.); 2 German Center for Mental Health (DZPG), Partner Site Bochum/Marburg, 44787 Bochum, Germany; 3 Cardiff University Centre for Human Developmental Science (CUCHDS), School of Psychology, Cardiff University, Cardiff CF10 3AT, UK; gersons@cardiff.ac.uk; 4 Department of Psychology, University of Toronto, Toronto, ON M5S 3G3, Canada; jessica.sommerville@utoronto.ca

**Keywords:** sleep, memory, early childhood, source monitoring, actigraphy

## Abstract

Sleep facilitates memory consolidation across the lifespan, but few studies have investigated underlying mechanisms in early development. The present study assessed the role of sleep timing after a social learning situation for action memory in seventy-six 4-year-old children and tested if source monitoring errors explain potential sleep effects (preregistration doi: 10.17605/OSF.IO/QSD2E). We employed a mixed-design combining an experimental within-subject manipulation of social interaction (high-vs.-low-collaboration condition) with an observational component measuring latency to nighttime sleep onset at T1. At T1, children participated in a social learning task scheduled at different times across the day to vary the interval between encoding and nighttime onset. Immediately following the task at T1, and again two weeks later, children’s memory for which actions had been performed (action memory) and who had performed them (agent memory, as an indicator of source memory) was assessed behaviorally. Sleeping behavior and the latency to nighttime sleep was assessed using actigraphy. Contrary to expectations, both action and agent memory were unrelated to latency to nighttime sleep onset after social learning and, in exploratory analyses, to sleep efficiency and sleep duration in the night following learning. Methodological aspects including the retention interval and task difficulty might have contributed to the unexpected results.

## 1. Introduction

During the first years of life, children spend over half of their lifetime asleep. Sleep plays a foundational role in physical growth, neuro-development, and cognitive functioning (Bathory & Tomopoulos, 2017; El-Sheikh & Sadeh, 2015; Mindell & Williamson, 2018). Crucially, sleep directly drives memory consolidation in early childhood. Both nighttime sleep and brief daytime naps that follow soon after information encoding enhance visuospatial recognition and memory retention in infants, toddlers, and preschool-aged children (e.g., Henderson et al., 2012; Kurdziel et al., 2013; Seehagen et al., 2015; Wilhelm et al., 2008; see Mason et al., 2021, for a review). In the present study, we tested how the timing of nighttime sleep onset after a learning event affects young children’s memory across a two-week retention interval.

Beyond supporting quantitative retention, sleep also brings about qualitative alterations in memory, including the emergence of false memories. False memories are commonly evaluated via the Deese–Roediger–McDermott (DRM) paradigm. Here, participants exposed to semantically related word lists frequently generate false memories by falsely recalling an unstudied, thematic “critical lure” (Stadler et al., 1999). While the extraction of semantic “gist” typically develops with age, sometimes leading to an increase in DRM false memories over childhood, the underlying vulnerability to these distortions is already evident in early development. Indeed, when tasks are adapted for younger cohorts, five-year-olds demonstrate no significant differences from seven-year-olds and adults in their rate of critical lure generation (Ghetti et al., 2002). This developmental susceptibility extends directly to source monitoring (determining the origin of a memory, i.e., whether an event actually happened or just imagined). Young children struggle more than adults to distinguish between real and imagined actions (e.g., executing vs. imagining a motor action; Foley & Johnson, 1985) and commit frequent source-attribution errors when competing memory traces share high situational, perceptual, or semantic similarity (Lindsay et al., 1991). Because source monitoring undergoes significant developmental improvement during the preschool years, young children are uniquely vulnerable to these similarities, showing a heightened susceptibility to false memories whenever experiential origins closely resemble one another.

Research in adults demonstrates that true and false memories undergo distinct consolidation trajectories during sleep. Utilizing the DRM paradigm, McDermott (1996) and D. G. Payne et al. (1996) and J. D. Payne et al. (2009) found that while the recall of verbatim learned words significantly decays over 24 to 48 h, the false recall of unstudied semantic targets (critical lures) remains stable or even increases. This dynamic suggests two potential, non-exclusive mnemonic pathways: either false memories decay at a significantly slower rate than veridical ones, or they are actively generated anew over the course of the retention interval as a product of structural memory reorganization. Stickgold and Walker (2013) explained this via a sleep-dependent “memory triage” mechanism: during post-encoding sleep, active consolidation shifts away from preserving vulnerable, episodic details and instead prioritizes extracting thematic commonalities across a broader semantic network. For example, during a delay, the specific, memory trace of having read the distinct words *bed*, *rest*, *awake*, and *tired* rapidly deteriorates, whereas the overarching “gist” or semantic schema of the collection is preferentially preserved and integrated into long-term storage. Consequently, when individuals are tested after a delay, this strengthened semantic schema actively drives the offline generation of a false memory, leading them to confidently misremember having been presented the unstudied but highly related word *sleep*. Recent literature also points to other factors impacting DRM effects such as task features (i.e., the number of words within a list and the type of memory test used; Newbury & Monaghan, 2019) and inter-individual factors such as learning performance (with increased false memories only in low-performers; Pardilla-Delgado & Payne, 2017).

Whereas adult sleep studies often focus on abstract linguistic paradigms such as learning word lists as in the DRM task, memory consolidation in childhood operates heavily within social contexts. Children learn through play and observation in natural contexts. Social learning is a key way of knowledge acquisition in the first years of life and can occur observationally or in direct collaboration with others (e.g., Barr & Hayne, 2003). Collaborative activities are defined by two or more agents coordinating complementary actions to achieve a shared intentional goal (Bratman, 1992). Collaboration can lead to better problem-solving in 6–7-year-olds (Fawcett & Garton, 2005), better writing skills (Boscolo & Ascorti, 2004), and planning skills in school-aged children (Gauvain & Rogoff, 1989).

To map the cognitive mechanisms driving these learning gains, developmental research has scrutinized children’s source monitoring of collaborative events. Foley and Ratner (1998) discovered that after a turn-taking collage task, four-year-olds disproportionately misattributed the experimenter’s actions to themselves. That is, they were more likely to state that they performed an action that was in fact carried out by the experimenter than the other way around (i.e., misattributing their own actions to the experimenter). This phenomenon is referred to as the “I-did-it” bias. This asymmetry occurs primarily when children actively monitor their partner and mentally simulate executing the partner’s step rather than when they simply observe the actions passively without a shared goal (e.g., Sommerville & Hammond, 2007). Ratner et al. (2002) demonstrated that this source-monitoring error is highly context-dependent, occurring significantly more in collaborative settings than in parallel, non-collaboration tasks. They proposed that the bias stems from internalization (Kristen et al., 2006): a process where a child represents the self as both the actor and observer of a partner’s action. Crucially, Ratner et al. noted that children exhibiting higher “I-did-it” errors were better in a categorization task at test, suggesting that the misattribution reflects a learning advantage from deeply cognitive engagement.

Expanding this idea, Sommerville and Hammond (2007) manipulated the degree of collaboration during a multi-step toy assembly task with three- and four-year-olds. They confirmed that high-collaboration (turn-taking) conditions yielded a significantly greater “I-did-it” bias in both free and cued recall than low-collaboration (observational) conditions. Strikingly, a higher “I-did-it” bias was related to better action memory, both immediately and four months later. Thus, the deliberate internalization of a partner’s actions stabilized action memory over time, even at the cost of precise source attribution.

The present study closely followed the methodology of Sommerville and Hammond (2007). We examined four-year-old children’s “I-did-it” bias (as a measure of source memory) and reconstruction performance (as a measure of action memory) both immediately after a collaborative learning event (T1) and two weeks later (T2). This within-subject design involved an experimental manipulation of the type of collaboration (high- vs. low-collaboration tasks) and an observational assessment of subsequent nighttime sleep onset latency on the evening of T1. Expanding previous research, we investigated the role of sleep timing after the learning event for the emergence of the “I-did-it” bias and for action memory. In high-schoolers, sleep boosted declarative memory only when occurring within a few hours after learning (Gais et al., 2006). Similar results were found in preschoolers, where the time elapsed before falling asleep (latency to nighttime sleep), significantly impacts memory; for instance, omitting an afternoon nap impaired recall in four-year-olds in ways that subsequent nighttime sleep cannot remedy (Kurdziel et al., 2013). Furthermore, night sleep following an evening learning event helped 8–11-year-old children extract explicit knowledge and boosted memory performance (Wilhelm et al., 2013). Consequently, analyzing the interval between T1 and nighttime sleep is essential to understanding memory changes across an extended delay as used here, specifically whether timely sleep leads to superior action memory compared to delayed sleep.

In terms of mechanisms, the post-learning sleep window may influence not only general action memory that is, retention, but also how children remember *who* performed an action. Sleep might consolidate a generalized action schema at the expense of specific details like the actor (Stickgold & Walker, 2013), or it may consolidate the “I-did-it” bias as children internalize others’ actions. Using a two-week interval, we aimed to find a balance between task difficulty (i.e., being easier closer to encoding) and feasibility for parents to partake in a two-session design with a fixed time in between. To investigate these possibilities, we examined individual variability in sleep-onset latency on the evening of T1, with T1 appointments scheduled at various times throughout the day to naturally vary latency. We used actigraphy to precisely track the time elapsed between the collaborative event and nighttime sleep, relating this latency to changes in both action memory and the “I-did-it” bias from T1 to T2. Based on previous studies, we had the following predictions (preregistration: https://doi.org/10.17605/OSF.IO/QSD2E):

(1) We predict that across both types of tasks (high-, low-collaboration), children will commit more “I-did-it” errors than “You-did-it” errors at T1 and T2.
(2) We predict that children will commit more “I-did-it” errors in high-collaboration than in low-collaboration tasks, both at T1 and T2.
(3) We predict that children will achieve higher reconstruction scores in high-collaboration tasks than in low-collaboration tasks at T1 and T2.
(4) We predict that at T1, occurrence of “I-did-it” errors and reconstruction scores for both types of tasks will be unrelated to latency to sleep onset at night after T1. In other words, we predict that children’s initial task performance will be unrelated to their sleep prior to the testing day (i.e., their naturally occurring sleep–wake cycle.
(5) We predict that latency to nighttime sleep onset after T1 will predict the development of “I-did-it” errors (T2 minus T1) such that shorter latencies will be associated with an increase in “I-did-it” errors.
(6) As our key hypothesis, we predict that shorter sleep onset latency on the night after T1 will predict higher T2 reconstruction scores, mediated by T2 “I-did-it” errors (where more errors relate to higher reconstruction scores). This indirect effect will be moderated by condition, such that high-collaboration tasks will yield more “I-did-it” errors than low collaborative tasks.

Furthermore, we exploratively examined relations between nighttime sleep variables (efficiency, duration) after T1, and “I-did-it” errors, reconstruction scores, and correct order of reconstruction at T2.

## 2. Materials and Methods

### 2.1. Participants

The final sample consisted of 76 children aged 3–4 years (*M_age_* = 47.93 months, *SD* = 1.78 months, range = 36 to 50 months, 39 girls). All children had German nationality. Most parents of the participating children had a university degree (72% of the mothers, 75% of the fathers). The majority of children had one or more siblings (51% had one sibling, 10% had two siblings, 3% had three siblings). The participating children were recruited via a database of the developmental psychology lab at Ruhr University Bochum. Data collection took place from February to December 2023. The study was approved by the Ethics Committee of the Faculty of Psychology at Ruhr University Bochum on 7 November 2022.

Our key hypothesis was H6. Hence, we determined the required sample size for mediation analysis. Based on Fritz and MacKinnon (2007), a sample size estimation for the mediation models yielded a required sample of 72 children (assuming a medium effect size of 0.39 for both α and β paths using a bias-corrected bootstrap at 80% power).

An additional 14 children were tested but excluded due to experimenter error (*n* = 1), due to the children not wanting to complete the tasks (*n* = 2), because they missed the second session (*n* = 10), or due to a technical failure (*n* = 1).

For analyses of latency to nighttime sleep, *n* = 8 children were excluded due to missing information on nocturnal sleep (i.e., having no diary sleep time or actigraphy). When no actigraphy data was available, sleep times from parent diaries were used and vice versa. For analyses of sleep measures derived from actigraphy (i.e., sleep efficiency and sleep duration), 11 children had to be excluded for missing actigraphy data.

### 2.2. Design

This study utilized a within-subject design combining an experimental manipulation with an observational component. The experimental factor consisted of condition type (high collaboration vs. low collaboration), while the observational factor was latency to nighttime sleep onset on the evening of T1 (with T1 appointments distributed throughout the day to naturally vary the time children spent awake until falling asleep in the evening). Concerning the T1 session scheduling, N = 22 children performed the encoding session prior to 12 p.m. while the other children were scheduled later. To control for this timing and for children who did nap after encoding, we also performed all analyses on sleep timing using the latency to next sleep (see Section 3). Memory performance, comprising agent memory and action memory, was assessed across a within-subjects factor of time (T1: after a 5 min break; T2: after a 2-week delay). To eliminate order and material effects, the allocation of the four toys to conditions, the presentation order of the conditions, and the identity of the initial builder (experimenter vs. child) were fully counterbalanced. For the agent memory task, the 24 images of the building steps were presented in one of three balanced sequences, pseudo-randomized to ensure that no more than two steps from the same toy and no two consecutive steps occurred in succession.

### 2.3. Material

#### 2.3.1. Stimuli

**Figure 1:**
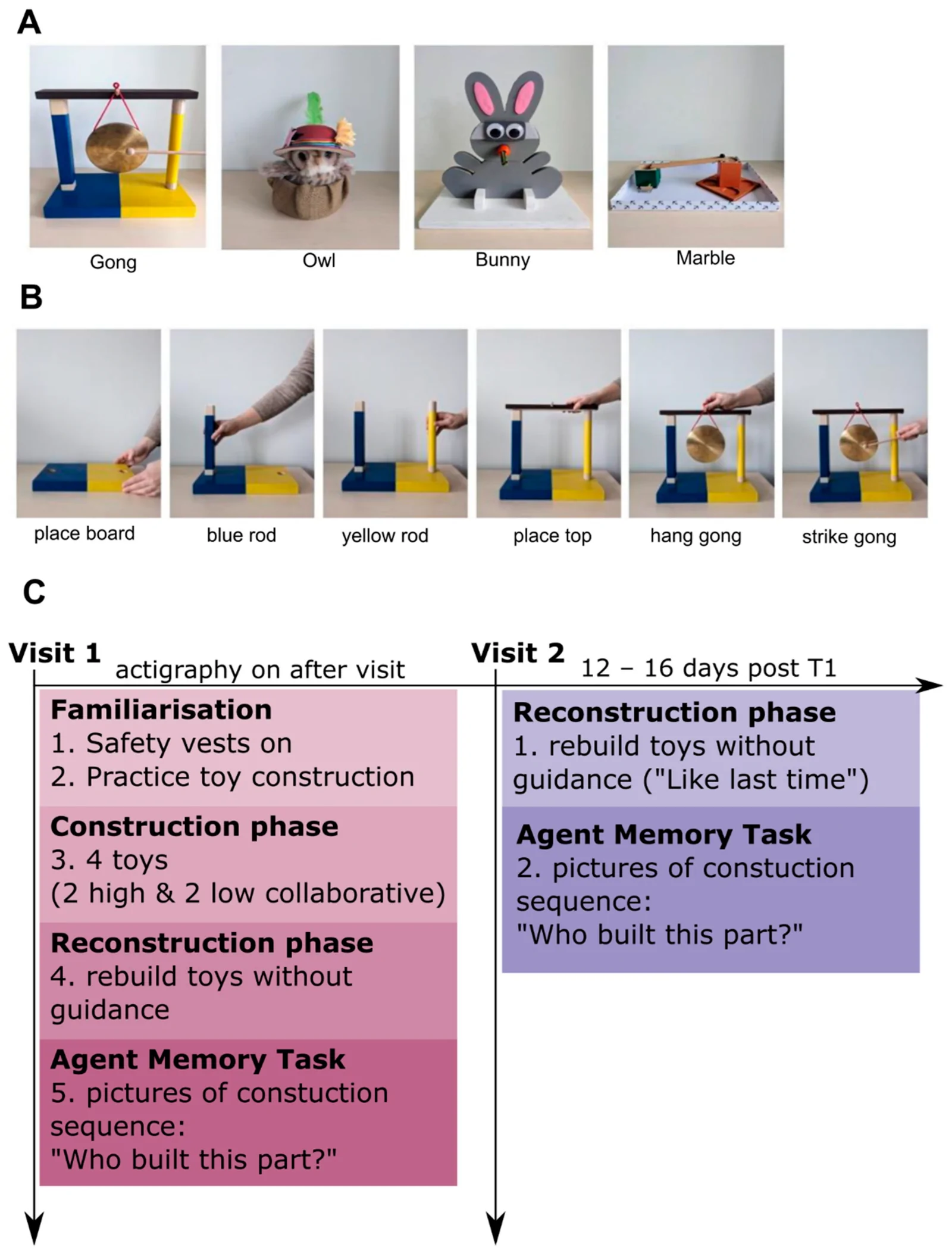
Task overview. (**A**) Depiction of the four toys in their assembled state. (**B**) Steps for the Gong task as an example for construction steps. (**C**) Session overview.

Four custom-built, six-step toys were adapted from Sommerville and Hammond (2007) (see Figure 1 for assembly steps). Two toys featured an enabling sequence where steps built sequentially on one another (gong, marble run), while two featured an arbitrary sequence with independent steps (owl, rabbit). A four-step Lego Duplo house served as a familiarization toy. Toy components were stored in individual boxes. For each toy, six laminated A5 photographs illustrated the assembly steps (24 photos total). These pictures guided assembly during the construction phase and served as visual prompts during the agent memory task to assess the “I-did-it” bias.

#### 2.3.2. Safety Vests as a Memory Cue

To reinstate context and facilitate source memory, both the child and experimenter wore a yellow safety vest during the construction and agent recall phases at T1 and T2. Prior to the agent memory task, memory for this context was verified by asking, “*What was different just now/last time from what we did together? Were we wearing a hat or a vest?*”

#### 2.3.3. Sleep Measurement

To record sleep/wake times, Actiwatches Micro Motionlogger (Ambulatory Monitoring) were used. These measure the child’s motor activity and use a specially developed algorithm to calculate the child’s sleep and wake times. The Sadeh Algorithm used in this study is based on the publication by Sadeh et al. (1994). Additional data on bedtime, sleep onset and nighttime awakenings was collected using a sleep and activity diary completed by the parent.

### 2.4. Procedure

#### 2.4.1. Visit 1

Upon arrival at the child lab at Ruhr University Bochum, the child participated in a warm-up phase using age-appropriate toys while the parent completed the informed consent form and questionnaires. Afterwards, the child’s consent was obtained verbally. Testing took place at a child-sized table positioned against a 160 cm gray partition wall to minimize distractions. The child sat directly at the table, the experimenter sat diagonally opposite, and the parent sat behind the child out of their direct line of sight. Materials were stored to the right of the experimenter. The session was recorded using two cameras (one side-view and one top-down view from the partition wall). See Figure 1C for a procedural overview.

**Familiarization phase.** The familiarization phase introduced the child to step-by-step toy construction using pictorial guides. First, both the experimenter and the child put on safety vests to establish a contextual cue for the later agent memory task. The experimenter then demonstrated the alternating, turn-taking procedure using a practice toy (“the house”). For the first step, the experimenter presented a picture, modeled the action using a component from the materials box, and pointed to the completed step. For the second step, the child was shown the corresponding picture and instructed to replicate it. This alternating process continued until the practice toy was fully assembled. Finally, the experimenter explained that for the upcoming toys, they would either continue taking turns or one person would build the entire toy alone.

**Construction phase.** In the construction phase, the experimenter and the child built the four toys according to the procedure described for the familiarization phase. Two of the toys were constructed in alternating steps (i.e., high-collaboration condition), whereas for the low-collaboration condition, one of the toys was built completely by the experimenter and one completely by the child while the other person observed the process. Both toys for the same condition were constructed after one another. If the child had difficulty with or performed a step incorrectly, the experimenter provided verbal support using standardized phrases. Once all the toys had been constructed, the experimenter and the child took off their safety vests. After this, there was a five-minute break in which the experimenter and the child stood up from the table and went to another small table to do some coloring or a jigsaw puzzle.

**Reconstruction phase.** After the 5 min break, the reconstruction phase began with the participants returning to their designated seating positions. The experimenter sequentially presented the boxes containing components for each of the four toys (ordered identically to the construction phase) and instructed the child to rebuild them without the step-by-step pictorial guides. Fully reconstructed toys were immediately removed from the table before presenting the next box. If a child signaled completion but the toy was incorrect or incomplete, the experimenter delivered up to two standardized prompts at 10-to-12 s intervals (“*You have a little time left. Build it exactly the way it was before*.”). If the toy remained incorrect 10 s after the final prompt, the experimenter removed it and advanced to the next trial.

**Agent memory task.** To assess memory of the construction phase, children were first asked if they had worn a hat or a safety vest during construction. To reinstate context, both participants put their safety vests back on. The experimenter then sequentially presented 24 pictures of the construction steps (six per toy), pointed to the relevant action, and asked: “*When we had the vests on and built the toys together, who built this part? Did you do it or did I?*” Afterwards, the child was given the actigraph to wear around their wrist until the next morning. The parents were paid the 10€ expense allowance.

#### 2.4.2. Visit 2

The second measurement point took place after 12–16 days. As at T1, the same experimenter received the family and spent a few minutes initially getting used to the room with the child, either drawing or playing. Then, the experimenter obtained the child’s consent for the procedure and started at the table in the same position as at T1.

**Reconstruction phase.** The procedure for the reconstruction phase at T2 was identical to the reconstruction phase at T1, except for the instruction “Build it again exactly as it was before.” was changed to “Build it again exactly as it was last time.” The order of the four toys also corresponded to the order of construction and reconstruction at T1.

**Agent memory task.** This task was identical to the procedure as described for T1. First, the question about the safety vest was asked as a reminder, and then the pictures of the 24 steps were presented again. Finally, the child’s level of ToM development was assessed but will be reported elsewhere. Afterwards, the child received a small gift and a participation certificate, and the parents were paid the 10€ expense allowance.

### 2.5. Data Coding and Reduction

Data form the video-recorded test sessions were coded offline using the INTERACT software tool (Version 18.03.20; Mangold International, Arnstorf, Germany). Children’s reconstruction performance was assessed via two percentage scores calculated per condition (high collaboration vs. low collaboration): a reconstruction score based on the number of steps correctly reconstructed on the first attempt (0–6 points per toy) and a sequence score based on the number of correctly ordered adjacent step pairs on the first attempt (0–5 points per toy; scoring terminated at the first out-of-sequence step). For agent memory recall, children attributed the 24 construction steps to either themselves or the experimenter, yielding raw totals (0–12 points) for I-did-it biases (erroneous self-attributions) and “You-did-it” biases (erroneous experimenter-attributions). These raw totals were converted into condition-specific percentage scores to accommodate the exclusion of individual toys in cases of procedural error. We computed “I-did-it” and “You-did-it” error percentages separately (i.e., for “I-did-it”: number of incorrect attribution to self/total number of steps in task). Inter-rater reliability was assessed on a subset of videos (*n* = 14) based on two independent raters. The intra-class correlation coefficient (ICC) for average random raters (ICC2k) was calculated in R using the “I-did-it” and “You-did-it” biases from T1 and T2 to assess the reliability of the ratings. The results (ICC2k = 0.97, 95% CI [0.96, 0.98], *F*(55, 55) = 44.58, *p* < 0.001) demonstrated high agreement (Greguras & Robie, 1998).

Actigraphy was used to measure latency to nighttime sleep onset (in minutes) following T1. For exploratory analyses, we assessed duration of nocturnal sleep and sleep efficiency during the night following T1. Actigraphy data was analyzed using Action-W software (version 02, Ambulatory Monitoring Inc., Ardsley, NY, USA) in 1 min epochs using zero-crossing mode. Sleep efficiency was calculated as the time asleep during the down interval (out of the total time in bed until children woke up).

## 3. Results

### 3.1. Confirmatory Analyses

**Figure 2:**
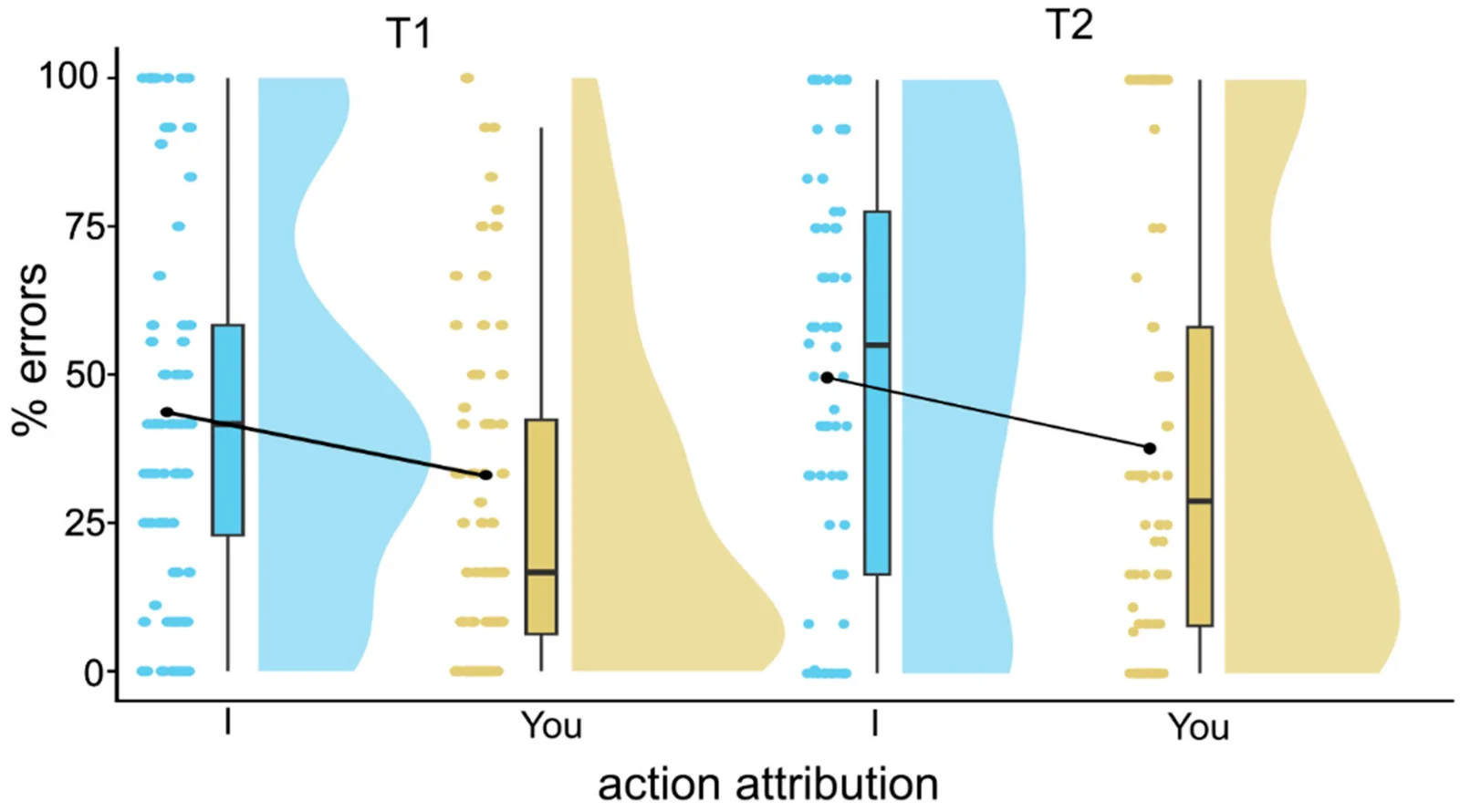
Effects of errors on day one and day two. Higher number of “I-did-it” errors (blue) compared to “You-did-it” (yellow) on day 1, higher numbers of “You-did-it” errors on day 2 compared to day 1. Dots reflect individual children.

To test H1, we conducted paired t-tests to compare the percentage of “I-did-it” errors and “You-did-it” errors at two measurement points (T1 and T2) across both high- and low-collaboration tasks. We computed “I-did-it” errors and “You-did-it” errors separately as the sum of the respective error type divided by the total number of performed actions multiplied by 100 to produce a percentage for “I” and “You” errors. At T1, the paired t-test revealed a significant difference between the percentage of “I-did-it” errors (*M* = 43.68, *SD* = 32.23) and “You-did-it” errors (*M* = 28.47, *SD* = 28.63; *t*(75) = 2.49, *p* = 0.014, d = 0.49) with more “I-did-it” errors (Figure 2). At T2, the paired t-test showed no significant difference between the percentage of “I-did-it” errors (*M* = 49.82, *SD* = 35.59) and “You-did-it” errors (*M* = 37.88, *SD* = 36.31; *t*(75) = 1.53, *p* = 0.128, d = 0.33). Thus, H1 had to be rejected partly. Since we found an effect for day one but not for day two we performed exploratory paired t-tests between “I-did-it” errors at T1 and T2 and one paired t-test between “You-did-it” errors at T1 and T2. Here, we find a significant effect for “You-did-it” errors only, with an increase from T1 to T2 (“I-did-it” T1 vs. T2: *t*(75) = −1.64, *p* = 0.103, d = −0.18; “You-did-it” T1 vs. T2: *t*(75) = −2.79, *p* = 0.006, d = −0.28; Figure 2).

**Figure 3:**
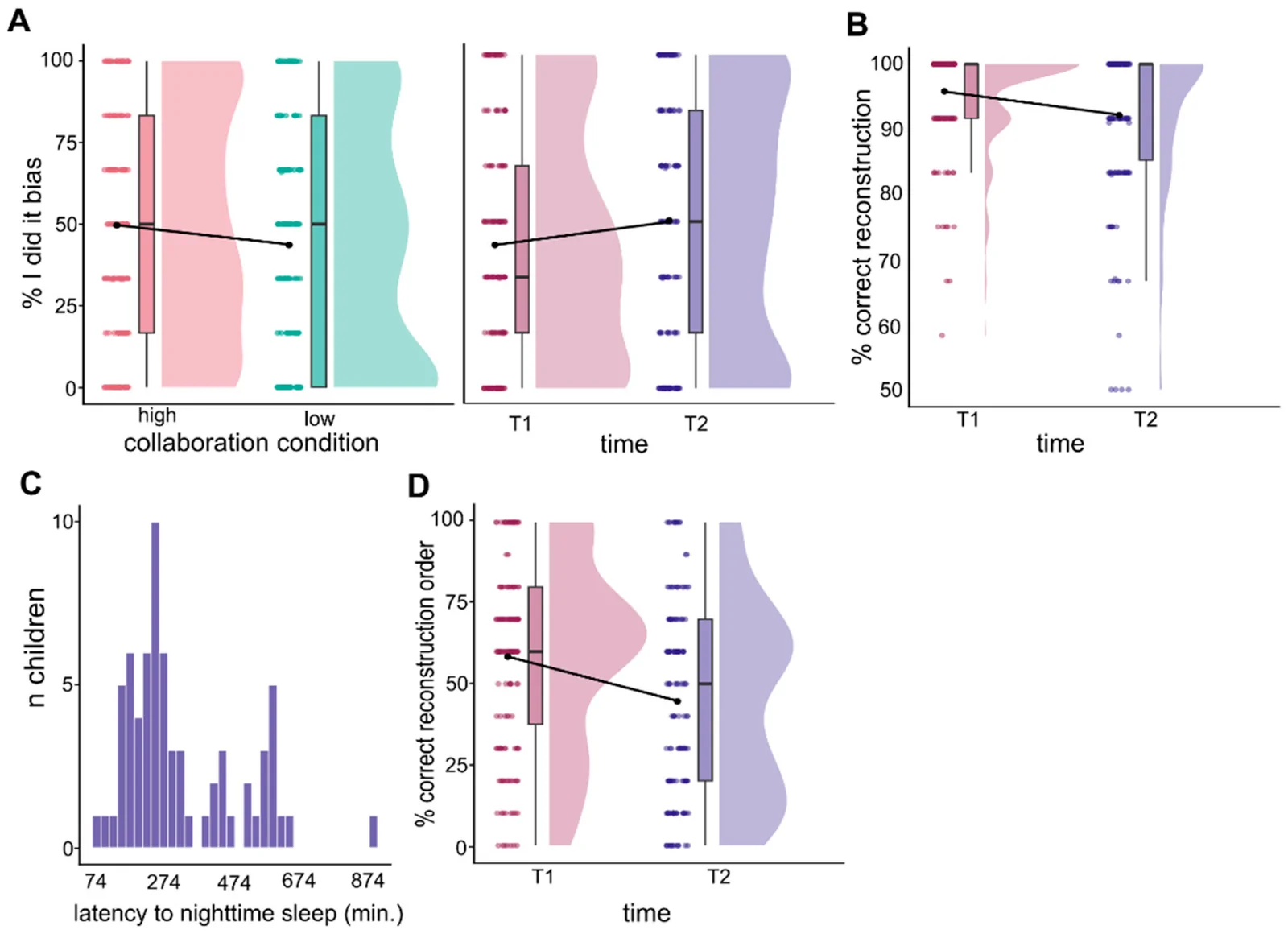
Effects of bias, time and collaboration condition. (**A**) Higher “I-did-it” bias in high-collaboration task and at T2. (**B**) Higher reconstruction scores at T1. (**C**) Distribution of latencies to nighttime sleep across children. (**D**) Better sequence memory (correct order) on day 1. Dots reflect individual children. High and low collaboration are reflected in coral (high) and mint (low). Time is reflected in pink for T1 and lilac for T2.

Next, we conducted a 2 (task type: high-, low-collaboration) × 2 (measurement point: T1, T2) repeated-measures ANOVA on the percentage of “I-did-it” errors. We found significant main effects of both time (*F*(1, 75) = 4.02, *p* = 0.048, η^2^_G_ = 0.010) and collaboration condition, (*F*(1, 75) = 6.12, *p* = 0.015, η^2^_G_ = 0.006) with a higher “I-did-it” bias for the high-collaboration task and a higher “I-did-it” bias on the subsequent day compared to the first day (Figure 3A). The interaction effect between time and collaboration condition was not significant (*F*(1, 75) = 0.003, *p* = 0.957, η^2^_G_ < 0.001).

For Hypothesis 3, we conducted a 2 (task type: high-, low-collaboration) × 2 (measurement point: T1, T2) repeated-measures ANOVA on reconstruction scores (expressed in percent). Again, we found a significant main effect of time (*F*(1, 74) = 11.97, *p* < 0.001, η^2^_G_ = 0.032; Figure 3B) with reconstruction scores being higher at T1 compared to T2. However, neither collaboration condition (*F*(1, 74) = 0.05, *p* = 0.815, η^2^_G_ < 0.001) nor the interaction of time and task type (*F*(1, 74) =0.094, *p* = 0.759, η^2^_G_ < 0.001) showed a significant effect.

As predicted, we did not find a relation between latency to nighttime sleep onset and behavioral outcomes on T1. To test this, we performed correlation analyses, which revealed no significant correlations (“I-did-it” errors in high-collaboration tasks: *r*(66) = 0.04, *p* = 0.736; “I-did-it” errors in low-collaboration tasks: *r*(66) = 0.05, *p* = 0.654; reconstruction scores in high-collaboration task: *r*(66) = −0.17, *p* = 0.155; reconstruction scores low-collaboration task: *r*(66) = 0.01, *p* = 0.923; *n* = 8 based on parent reports). This analysis confirmed that there were no variations in short-term source and agent memory between children who participated at different times during the day, which could have affected performance at T2.

Next, we tested whether latency to nighttime sleep onset after encoding would predict the development of “I-did-it” errors (T2–T1). Thus, we conducted linear regression analysis with latency to nighttime sleep onset (*M* = 324.72, *SD* = 170.16; see Figure 3C or the distribution of latencies in our sample; N = 8 based on parent reports) as the predictor and the difference score of “I-did-it” errors as the outcome. Contrary to our prediction, we did not find a significant relation between the bias and latency to nighttime sleep onset (*t*(66) = 0.38, *p* = 0.701, R ^2^= 0.002). Thus, H5 had to be rejected.

To test Hypothesis 6, a moderated mediation analysis was conducted using the mediation package (Tingley et al., 2014) in R with 5000 bootstrap samples to evaluate whether shorter latency to nighttime sleep onset after encoding at T1 predicted higher reconstruction scores at T2 via the percentage of “I-did-it” errors at T2, and whether this indirect path was moderated by the collaboration condition (high vs. low). First, a linear regression model was constructed to test if task collaboration moderated the effect of latency to nighttime sleep onset on the percentage of “I-did-it” errors. The main effect of sleep latency (β = 0.027, *t*(132) = 1.33, *p* = 0.185) and the main effect of task collaboration (β = 11.51, *t*(132) = 0.83, *p* = 0.40) were not significant. Crucially, the interaction effect between sleep latency and task collaboration type on bias errors was non-significant (β = −0.01, *t*(132) = −0.33, *p* = 0.735). Next, a second linear regression model was estimated to predict T2 reconstruction scores. The results showed that the effect of “I-did-it” errors on reconstruction scores (β = −0005, *t*(132) = −0.20, *p* = 0.836; *n* = 8 based on parent reports) and the direct effect of latency to nighttime sleep onset on reconstruction scores (β = −0.007, *t*(132) = −1.28, *p* = 0.20) were not significant.

To fully evaluate the conditional process, the Average Causal Mediation Effects (ACME) and Average Direct Effects (ADE) were examined across both levels of the task collaboration moderator. For low-collaboration tasks, the conditional indirect effect of latency to nighttime sleep onset on reconstruction scores via “I-did-it” errors was non-significant, ACME w < 0.001, 95% CI [−0.002, 0.001], *p* = 0.872 (*n* = 8 based on parent reports). The average direct effect for high collaboration tasks was also non-significant, ADE = −0.007, 95% CI [−0.021, 0.003], *p* = 0.201, yielding a non-significant total effect, Total Effect = −0.008, 95% CI [−0.021, 0.003], *p* = 0.186. For low-collaboration tasks, the conditional indirect effect remained non-significant, ACME < 0.001, 95% CI [−0.002, 0.002], *p* = 0.851. The average direct effect within high collaboration tasks was likewise non-significant, ADE = −0.007, 95% CI [−0.021, 0.003], *p* = 0.196, resulting in a non-significant total effect, Total Effect = −0.008, 95% CI [−0.021, 0.003], *p* = 0.182. Because the bootstrap confidence intervals for the indirect effects contained zero at both levels of the moderator, and the conditional effects did not meaningfully differ, Hypothesis 6 was not supported.

Please note that eight children answered the memory question (“*What was different just now/last time from what we did together? Were we wearing a hat or a vest?*”) incorrectly. We thus performed all analyses again on children who reported the memory question correctly. With this sample, the pattern of the results remained the same except for H1 (which reveals a difference between “I-did-it” errors and “You-did-it” errors at T1 on a trend-level while being significant when using the full sample: *t*(67) = 1.90, *p* = 0.060, *d* = 0.40). In addition, please note that *n* = 8 children took a daytime nap between participating in T1 and starting their nighttime sleep that evening. In the main analyses we did not consider these children separately due to our focus on nighttime sleep. However, as the main analyses ignored their napping, we re-performed the analyses and included these eight children’s latency to nap onset (instead of latency to nighttime sleep onset, i.e., latency to next sleep). The results remained unchanged.

### 3.2. Exploratory Analyses

Next, we conducted exploratory analyses to investigate the effects of sleep efficiency (*M* = 91.39, *SD* = 6.38) and sleep duration (in minutes; *M* = 575.51, *SD* = 69.43) on “I-did-it” bias and reconstruction scores at T2 using linear regression models using the actigraphy data. We found no effects across all of these exploratory analyses (sleep efficiency on bias: *t*(49) = 0.44, *p* = 0.663, R^2^ = 0.003; sleep efficiency on reconstruction scores: *t*(49) = 1.14, *p* = 0.261, R^2^ = 0.025; sleep duration on bias: *t*(49) = 0.31, *p* = 0.757, R^2^ = 0.001; sleep duration on reconstruction scores: *t*(49) = 0.74, *p* = 0.466, R^2^ = 0.010; actigraphy only).

Finally, we conducted an exploratory 2 (time: T1, T2) × 2 (task type: high, low) repeated-measures ANOVA on the accuracy of correct order recall. As we found reconstruction scores to be quite high, we chose to analyze a more difficult task level for the children by investigating the accuracy of the order of the task steps. We found a significant main effect of time (*F*(1, 75) = 19.60, *p* < 0.001, η^2^_G_ = 0.053; Figure 3D) with a decrease in correct order recall on day 2 compared to day 1. We found no significant effect of task type (*F*(1, 75) = 0.09, *p* = 0.755, η^2^_G_ = 0.0003) and no interaction (*F*(1, 75) = 1.10, *p* = 0.289, η^2^_G_ = 0.003). In addition, we conducted a linear regression on correct order scores at T2 using latency to nighttime sleep onset as a predictor. However, we did not find a significant relation (*t*(66) = 0.628, *p* = 0.532, R^2^ = 0.005).

## 4. Discussion

In this preregistered study, we tested if latency to nighttime sleep after participation in social learning tasks predicted 4-year-olds’ action memory (measured by reconstruction performance) and whether this mnemonic benefit stemmed from sleep-related increases in the “I-did-it” bias as an indication of source memory. In accordance with our predictions, children were more likely to exhibit the “I-did-it” bias in a high-versus-low-collaboration learning context. Partly confirming our prediction, on T1 only, children were more likely to show an “I-did-it” bias than a “You-did-it” bias. Contrary to our predictions, action memory did not differ between low- and high-collaboration tasks, and a shorter latency to nighttime sleep onset after learning did not predict better action memory two weeks later. In addition, there was neither evidence of an effect of latency to nighttime sleep onset on the emergence of the “I-did-it” bias, nor of a mediating role of this bias in the relationship between sleep onset latency and long-term action memory.

A growing body of literature shows that sleep after a learning experience facilitates memory consolidation from infancy to adulthood (e.g., Backhaus et al., 2008; Horváth et al., 2018; Talamini et al., 2008). In early childhood, both naps and nighttime sleep are integral for long-term retention (Souabni et al., 2025). Unexpectedly in the present study, neither latency to nighttime sleep onset nor sleep efficiency or sleep duration predicted action memory. Several methodological factors might explain these null findings, indicating potential boundary conditions for sleep effects on memory in early childhood.

First, the active system consolidation account suggests that sleep plays a crucial role in transferring recently encoded memories from a temporary store, the hippocampus, to neocortical networks for integration and permanent storage (Diekelmann & Born, 2010). Conceivably, capacity of the hippocampus is still limited in infancy, necessitating more frequent sleep periods for transfer of memories to the neocortex than is required in adulthood (Seehagen et al., 2015). Without timely sleep, recently encoded memories might be lost after hippocampal encoding capacity has been exhausted. Conversely, maturation of hippocampus-dependent memory networks has been suggested to yield more efficient memory storage and slower build-up of homeostatic sleep pressure (Spencer & Riggins, 2022). Hence, with increasing age, children might tolerate increasingly long intervals between information encoding and sleep onset without mnemonic consequences. Children in our sample were around 4 years old and might have been fully capable of retaining recently encoded memories across an entire day (if assessed in the morning) before sleep-dependent consolidation supported transfer to long-term storage at night. In support of this idea, some studies with older children (Backhaus et al., 2008) and adults (Petzka et al., 2023) indicated that delayed sleep onset after a learning experience can benefit memory. Considering this, the present study design might have yielded different results with younger children who are reliant on more frequent sleep periods for memory consolidation. Future studies could systematically vary sleep onset latency between groups of differently aged children and study memory outcomes after sleep.

Second, and alternatively to the first explanation, it is possible that we missed a short-term effect of sleep on action memory that was initially present for example, on the day following T1. In some previous studies with adults, sleep effects on memory washed out such that they were initially observable and then faded over time (e.g., Schönauer et al., 2015). However, this pattern is not consistently found in children and adolescents. Instead, timely sleep after a learning experience seems to have lasting consequences for memory in development (e.g., Kurdziel et al., 2013; Lemos et al., 2014; Seehagen et al., 2015; Williams & Horst, 2014). From a theoretical perspective, it will be informative to test retention after multiple delays (e.g., Williams & Horst, 2014). A potential caveat in this approach is that either large samples are needed to test different children after different delays, or that potential practice effect could complicate interpretation of long-term performance if the same children are tested multiple times. From an applied perspective, we suggest that the current design offers relevant information on how children remembered a one-off learning experience after a substantial delay as a function of its timing in relation to subsequent sleep.

Third, sleep-dependent memory processing seems to partially depend on task difficulty (Diekelmann et al., 2009). In other words, if there are floor or ceiling effects in task performance, sleep effects on memory are difficult or impossible to document (e.g., Hermesch et al., 2025). In the present study, children performed near ceiling levels on action memory, especially at T1. However, whereas this might have obscured potential sleep effects in the main analyses, the explorative analyses considered correctly ordered sequences. Here, performance was lower, but latency to nighttime sleep onset did still not predict children’s performance. Hence, task difficulty alone is an unlikely explanation for the absence of sleep effects in the present study, but it might have been a contributing factor.

As expected, children were more likely to show the “I-did-it” bias in a high-versus-low-collaboration setting (Sommerville & Hammond, 2007). Presumably the bias emerges in collaborative contexts because children imagine themselves performing their social partner’s actions, hence internalizing the social partner’s actions. Partly in line with expectations, children were significantly more likely to commit “I-did-it” errors than “You-did-it” errors at T1 but not at T2. There was an increase in “I-did-it” bias across the retention interval, which presumably stemmed from a weakened memory trace of the original event after a 2-week delay. Note that we calculated “I-did-it” errors and “You-did-it” errors separately. The present study was closely based on the procedure of Sommerville and Hammond with two exceptions. Due to our focus on sleep-dependent memory, we tested action memory before agent memory whereas the order was reversed in Sommerville and Hammond. It is possible that this procedural change was consequential because the reconstruction task might have reminded children who had performed which action initially, thereby influencing the occurrence of the “I-did-it” bias. In addition, we used a within-subject design rather than a between-subject design for the low- vs. high-collaboration tasks. It is possible that children confused the conditions after the delay.

Contrary to expectations, and central to our study goals, the “I-did-it” bias was unrelated to sleep onset and memory performance. In a study by Pardilla-Delgado and Payne (2017) with adult participants using the DRM paradigm, retention of true memories was supported by sleep that occurred soon after learning. Timely sleep increased false memory only in low performers. In addition, there was a negative correlation between slow-wave-sleep (SWS) and false memory which might indicate a detrimental effect of sleep for gist processing. Whereas the present paradigm clearly differed from the DRM, effects of task difficulty and sleep architecture could be relevant to explain why the “I-did-it” bias did not vary as a function of sleep timing. Overall, children achieved high reconstruction scores, hence any potentially facilitative effects of the “I-did-it” bias might have not been detected. In addition, children show more SWS in their nighttime sleep than adolescents and adults (Scholle et al., 2011). If SWS suppresses abstraction processes like the findings from Payne et al. suggest, it is possible that it helps prevent source monitoring errors such as the “I-did-it” bias. However, in contrast to these considerations, the broader literature suggests that SWS might support abstraction processes (Keeble et al., 2025). This would lead to the hypothesis that more SWS is related to more source monitoring errors. Given that actigraphy data do not allow differentiation of sleep stages, any interpretation of the present results invoking sleep architecture remains speculative. Future studies should investigate the relations of different sleep stages (e.g., assessed via polysomnography) related to source memory effects in children using technology that offers more insights into the overall sleep architecture such as light and deep sleep (De Sena et al., 2024).

The present study has several limitations. First, children were tested on the same objects at T1 and T2. As our main goal was to assess the “I-did-it” bias and reconstruction memory over time, the same four toys were evaluated at both T1 and T2. One consequence of this design is that reconstruction of the toys at T1 may have induced a practice effect, thereby improving overall memory performance at T2. Future studies should include a parallel set of constructed items that are constructed at T1 and only tested for the first time at T2. Second, as indicated above, task difficulty was low, which might have impaired the ability to detect a beneficial effect of sleep. Third, a small minority of children (*n* = 8) napped between participating in T1 and falling asleep for the subsequent night. Future studies should control napping behavior after a learning event and collect information on habitual napping of participants. Fourth, the study included a single age-group. Given rapid changes in sleeping behavior and memory across infancy and early childhood, considering their relation across different ages within a given study is desirable.

## 5. Conclusions

In conclusion, the present study failed to reveal sleep effects on long-term action memory in four-year-olds. This was likely due to methodological factors including task difficulty, selected retention interval, and reliance on naturally occurring variation in latency to nighttime sleep onset. Identifying boundary conditions for sleep effects on memory as well as studying potential underlying mechanisms across the lifespan can help understand what, when, and why sleep supports memory consolidation and related cognitive processes.

## Data Availability

The original data presented in the study as well as the generated notebooks and code are openly available at the Open Science Framework (OSF) at https://osf.io/hfgzn/overview (accessed on 3 August 2026).
